# Bisphosphonate zoledronic acid blocks secretory autophagy and inhibits bone resorptive functions in osteoclasts

**DOI:** 10.1080/15548627.2026.2691880

**Published:** 2026-06-28

**Authors:** Sol Kim, Vadim Goldshteyn, Atsushi Arai, Eun-Bin Bae, Julian Whitelegge, Ki-Hyuk Shin, No-Hee Park, Reuben H. Kim

**Affiliations:** a The Shapiro Family Laboratory of Viral Oncology and Aging Research, UCLA School of Dentistry, Los Angeles, CA, USA; b Division of Preventive and Restorative Sciences, UCLA School of Dentistry, Los Angeles, CA, USA; c Section of Biosystems and Function, UCLA School of Dentistry, Los Angeles, CA, USA; d Institute for Oral Science, Department of Orthodontics, Matsumoto Dental University, Nagano, Japan; e The Pasarow Mass Spectrometry Laboratory, the Jane and Terry Semel Institute for Neuroscience and Human Behavior, UCLA David Geffen School of Medicine, Los Angeles, CA, USA; f UCLA Jonsson Comprehensive Cancer Center, Los Angeles, CA, USA; g UCLA David Geffen School of Medicine, Los Angeles, CA, USA

**Keywords:** Autophagy, bisphosphonate, osteoclasts, prenylation, RAB7

## Abstract

Bisphosphonates (BPs) are the most widely used anti-resorptive agents and first-line drugs for managing bone-related diseases, such as osteoporosis, Paget disease of bone, and bone metastatic cancer. BPs are known to inhibit osteoclasts’ functions, and recent studies have highlighted the importance of macroautophagy/autophagy in osteoclasts. However, the involvement of autophagy in BP-mediated inhibition of osteoclast functions remains unclear. In this study, we showed that BPs inhibit the bone resorptive functions of osteoclasts by blocking autophagy. At the non-apoptotic doses, zoledronic acid (ZOL) inhibited autophagy by blocking autophagic flux and delaying the degradation of autophagy-related proteins. ZOL also prevented the cleavage and secretion of secretory proteins such as CTSK, ACP5/TRAP, and MMP9 essential for bone resorption. Mechanistically, ZOL inhibits the prenylation of the RAB7 small GTPase, a key protein that is required for autolysosome formation. *In vivo* studies showed that osteoclast-specific *rab7* conditional knockout mice exhibited osteopetrotic phenotypes. These findings provide insights into how BPs disrupt osteoclast function by blocking autophagy and suggest that targeting autophagy in osteoclasts could be a potential therapeutic approach for bone-related diseases.

**Abbreviations**: ACP5/TRAP: acid phosphatase 5, tartrate resistant, ATG: autophagy related, 54 BafA1: bafilomycin A1, BECN1: beclin 1, BMM: bone marrow-derived macrophage, CQ: 55 chloroquine, CSF1/M-CSF: colony stimulating factor 1, CTSK: cathepsin K, CTX-I: C-terminal 56 telopeptide of type I collagen, FPPS: farnesyl pyrophosphate synthase, GFP: green fluorescent 57 protein, GGTI: geranylgeranyltransferase I inhibitor, LAMP1: lysosome associated membrane 58 protein 1, MAP1LC3/LC3: microtubule associated protein 1 light chain 3, MMP9: matrix 59 metallopeptidase 9, N-BPs: nitrogen-containing bisphosphonates, P1NP: procollagen type I N- 60 terminal propeptide, PGGT1B: protein geranylgeranyl transferase type I subunit beta, RABGGTB: 61 Rab geranylgeranyltransferase subunit beta, RFP: red fluorescent protein, SQSTM1/p62: 62 sequestosome 1, TNFSF11/RANKL: TNF superfamily member 11, ZOL: zoledronic acid,

## Introduction

Bisphosphonates (BPs) are the most widely used anti-resorptive agents in managing bone-related diseases, such as osteoporosis, Paget disease of bone, and bone metastases [1]. Two mechanistic classes are recognized. Non-nitrogen BPs are metabolized to non-hydrolyzable ATP analogs that are cytotoxic to osteoclasts. Nitrogen-containing BPs (N-BPs), including zoledronic acid (ZOL), inhibit farnesyl pyrophosphate synthase (FPPS) in the mevalonate pathway, depleting isoprenoid lipids required for prenylation of small GTPases. In osteoclasts, at the cellular level, they inhibit vesicular trafficking and cytoskeletal organization at the ruffled border. Apoptosis is known to be one of the most common cellular responses to BPs in osteoclasts [2]. Clinically, ZOL is among the most potent N-BPs; it binds avidly to hydroxyapatite, is taken up by osteoclasts during bone resorption, and suppresses bone turnover. While these upstream mechanisms are well established, the precise molecular mechanisms underlying the effect of N-BPs on osteoclasts’ function are not yet fully elucidated.

Osteoclasts are differentiated from osteoclast precursor cells such as bone marrow-derived macrophages (BMMs) upon stimulation by TNFSF11/RANKL (TNF superfamily member 11) [3]. Mature osteoclasts are multi-nucleated, lysozyme-rich, and polarized cells with ruffled borders through which the cells release critical enzymes, such as CTSK (cathepsin K), ACP5/TRAP (acid phosphatase 5, tartrate resistant), or MMP9 (matrix metallopeptidase 9), required for bone resorption. Initially synthesized as pro-forms, these secretory proteins are further processed to their cleaved forms for their activation and secretion [4,5].

Recent studies have shown that autophagy plays a key role in osteoclast biology [6–12]. Autophagy is an essential cellular process activated under stressful conditions (e.g., starvation) to form autophagosomes, in which organelles are degraded and recycled for cell survival [13]. Emerging evidence suggests that autophagy, a cellular recycling process, can interact with protein secretion pathways, known as “secretory autophagy,” for functions beyond just cell survival [14–16]. Previous studies showed that autophagy becomes activated during osteoclast differentiation and plays a significant role in mediating the bone resorption functions of osteoclasts [8–10,17,18]. However, whether N-BPs play a role in autophagy during osteoclast differentiation and function remains elusive.

This study aimed to examine the effects of N-BPs on autophagy in osteoclasts. We show that ZOL, at the non-apoptotic concentrations (1 µM), impairs bone-resorptive function by inhibiting autophagy and disrupting the processing of osteoclast-secreted proteins, including CTSK, ACP5/TRAP, and MMP9. These findings refine our understanding of how ZOL acts on osteoclasts and identify secretory autophagy as a key mechanism underlying its antiresorptive efficacy.

## Results

### ZOL impairs autophagic flux during osteoclast differentiation without inducing apoptosis

To investigate the role of BPs such as zoledronic acid (ZOL) on osteoclasts at low doses, bone marrow-derived macrophages (BMMs) were treated with 1 and 5 µM of ZOL for 3 days. Our results revealed that ZOL treatment did not elicit apoptotic responses in these cells (Figure S1A-C). Since autophagy has been reported to play a role in osteoclastogenesis without inducing cell death [8–10], we used this non-apoptotic dose (1 µM) and examined whether BPs impact autophagy during osteoclast differentiation. Our results showed that, when treated with this dose, an increase in MAP1LC3/LC3 (microtubule associated protein 1 light chain 3)-II level was observed in both BMMs and RAW264.7 cells during TNFSF11/RANKL-induced differentiation at this non-apoptotic concentration (Figure 1A,B). When autophagic flux was assessed using bafilomycin A_1_ (BafA1), a lysosomal acidification inhibitor that blocks autophagic degradation [19], autophagic flux was significantly suppressed in ZOL-treated cells compared to untreated controls (Figure 1C,D). Autophagic proteins undergo degradation via the autophagy pathway [20], and the inhibition of autophagy blocks the degradation of ATG (autophagy related) proteins [21]. To investigate this further, we examined ATG protein levels and observed that these proteins remained substantially elevated even after 5 days of ZOL treatment (Figure 1E). Interestingly, the expression of autophagy-related genes such as *Becn1* (beclin 1) and *Sqstm1/p62* (sequestosome 1) was significantly reduced following ZOL treatment, indicating that increased protein levels by ZOL is not due to induction of gene expression but inhibition of protein degradation (Figure 1F,G). To visualize autophagic flux directly, BMMs were transduced with a retrovirus encoding RFP-GFP-LC3 (GFP: green fluorescent protein, RFP: red fluorescent protein) and treated with either ZOL or BafA1. Confocal microscopy demonstrated increased GFP:RFP puncta ratios in ZOL-treated cells, a pattern resembling that observed in BafA1 treatments, which are known to inhibit autophagolysosomal function (Figure 1H,I). This suggests that ZOL disrupts autophagic flux by blocking autophagosome degradation in lysosomes. Consistently, western blot analysis of BMMs treated with TNFSF11/RANKL exhibited reduced expression of autophagy-related proteins (SQSTM1, BECN1, ATG4B, ATG7), while autophagy inhibitors, BafA1 and chloroquine (CQ), as well as ZOL caused accumulation (Figure 1J). Together, these findings demonstrated that ZOL impairs autophagy during osteoclast differentiation by inhibiting autophagic flux.

**Figure 1.**
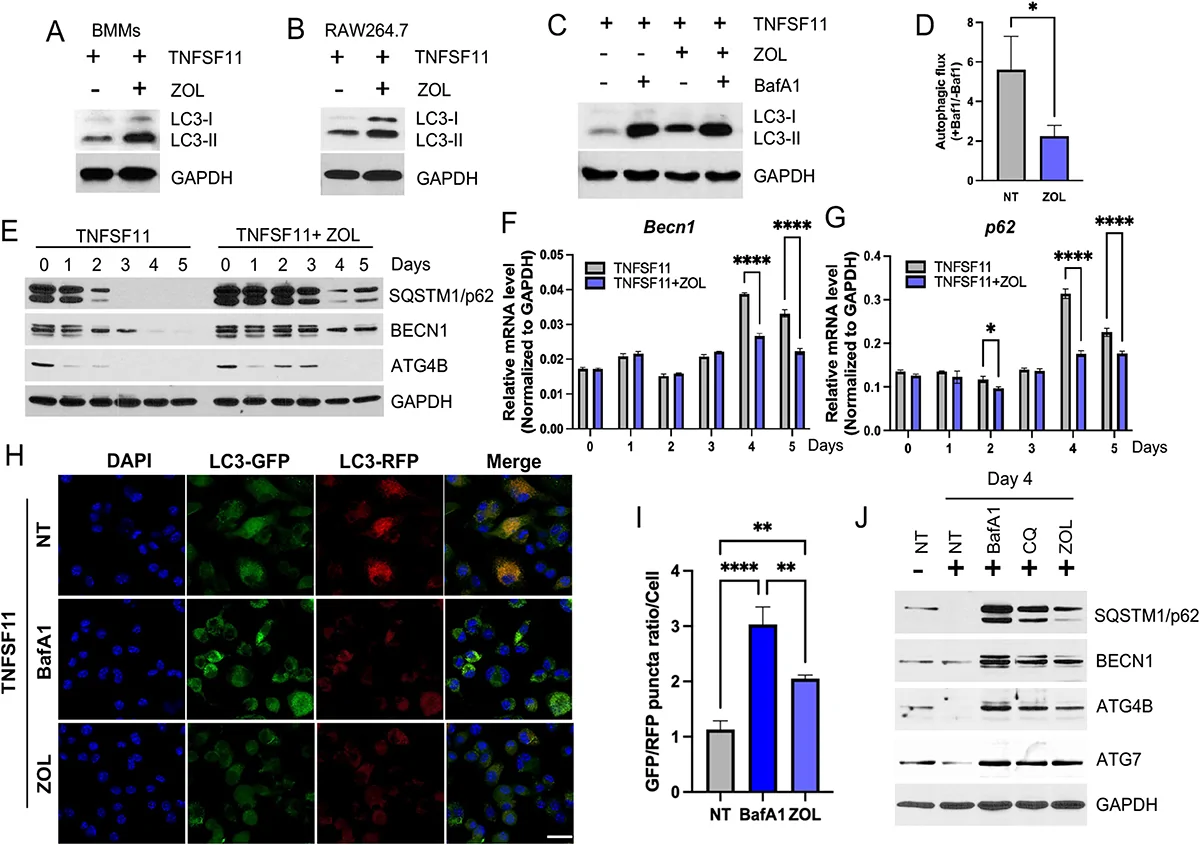
ZOL impairs autophagic flux during osteoclast differentiation. (A) BMMs were cultured with CSF1/M-CSF and TNFSF11/RANKL (100 ng/ml) and treated with or without ZOL (1 μM). Western blotting for MAP1LC3/LC3 and GAPDH. (B) RAW264.7 cells were induced with TNFSF11/RANKL (100 ng/ml) and treated with or without ZOL (1 μM). Western blotting for MAP1LC3/LC3 and GAPDH. (C) Western blotting for MAP1LC3/LC3 and GAPDH treated with or without ZOL (1 μM) in the presence of BafA1 (10 nM) in BMMs with CSF1/M-CSF and TNFSF11/RANKL (100 ng/ml). (D) Quantification of MAP1LC3/LC3 (*n* = biological triplicates). (E) Western blotting of SQSTM/p62, BECN1, ATG4B, and GAPDH with or without ZOL in BMMs for different time points. qRT-PCR for *Becn1* (F) and *Sqstm/p62* (G) in BMMs after inducing osteoclast differentiation. (H) BMM cells were infected with retrovirus RFP-GFP-LC3 and further treated with BafA1 (10 nM) or ZOL (1 μM) for 16 h followed by fluorescence microscopy. Nuclear counterstaining (DAPI, blue). NT: not treated. Scale bars: 20 μm. (I) Quantification of GFP:RFP puncta ratio of MAP1LC3/LC3. *n* = 3 wells; in each well, ~40–50 cells were quantified and averaged to yield one per-well mean. (J) Western blotting of SQSTM/p62, BECN1, ATG4B, ATG7, and GAPDH in BMMs treated with BafA1 (10 nM), CQ (50 nM), or ZOL (1 μM) for 4 days. Graphs show the mean ±SEM (D) or SD (F, G, and I). *p < 0.05, **p < 0.005, ****p < 0.0001. ns, not significant. Unpaired t-test (D). One-way ANOVA with Tukey’s multiple comparison test (I), two-way ANOVA with Sidak multiple comparison tests (F and G).

### ZOL inhibits the maturation of CTSK, ACP5/TRAP, and MMP9 in osteoclasts

Osteoclasts facilitate matrix degradation by secreting cleaved (active) CTSK, ACP5/TRAP, and MMP9 into the resorption lacuna [22,23]. Next, we investigated whether BPs played a role in the maturation and secretion of these proteins. When treated with a non-apoptotic dose of ZOL, a significant decrease in the conversion of ACP5/TRAP, CTSK, and MMP9 into their mature forms was observed during osteoclast differentiation (Figure 2A–C). Such inhibition by ZOL occurred in a dose-dependent manner, as demonstrated by ACP5/TRAP western blot (Figure 2D). Similarly, the activities and levels of the secreted CTSK, ACP5/TRAP, and MMP9 in the supernatants were decreased upon ZOL treatment (Figure 2E–G). Moreover, treatment with BafA1 and CQ suppressed the conversion of pro-enzymes into active enzymes for CTSK, MMP9, and ACP5/TRAP (Figure 2H). To explore whether fusion of autophagosomes with lysosomes occurs during osteoclastogenesis, we examined the colocalization of MAP1LC3/LC3 (an autophagosome marker) and LAMP1 (lysosome associated membrane protein 1; a lysosome marker). The results showed that MAP1LC3/LC3 was colocalized with LAMP1 during osteoclast differentiation, whereas the colocalization was significantly diminished when treated with ZOL, suggesting that ZOL inhibits the autophagosome pathway (Figure S1D and E). To further confirm this, we examined the colocalization of MAP1LC3/LC3 and RAB7, a marker of late endosomes and autophagosomes. In osteoclast differentiation, MAP1LC3/LC3 and RAB7 were well-colocalized, whereas this colocalization was disrupted by ZOL (Figure S1F and G). These findings suggest that ZOL inhibits the maturation and secretion of ACP5/TRAP, CTSK, and MMP9 by blocking autophagy at the level of autophagosome-lysosome fusion.

**Figure 2.**
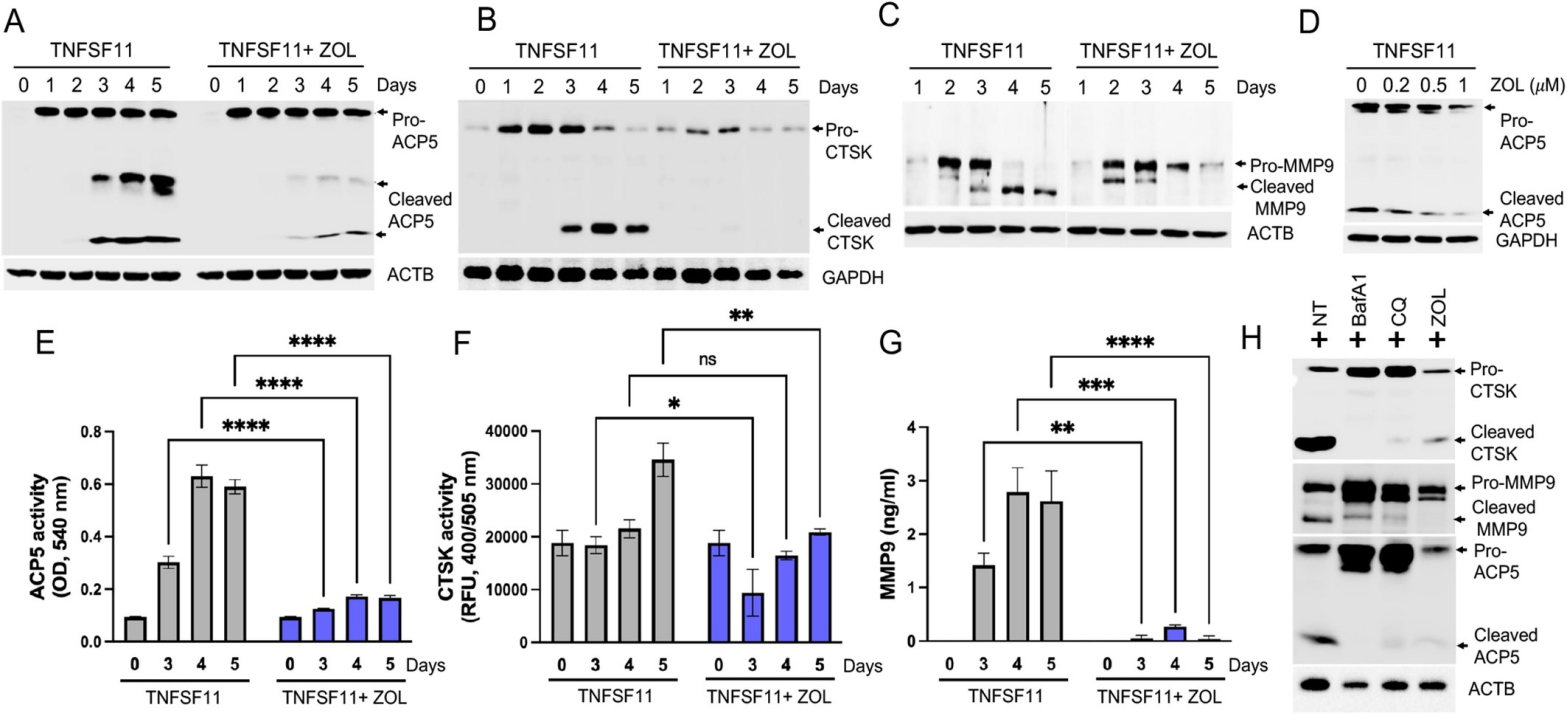
ZOL inhibits the maturation of CTSK, ACP5/TRAP, and MMP9 in osteoclasts. (A, B, C) Western blotting for CTSK, ACP5/TRAP, and MMP9 during osteoclast differentiation in BMMs treated with or without ZOL (1 μM). (D) Western blotting for ACP5/TRAP during osteoclast differentiation in BMMs treated with different doses of ZOL. (E) ACP5/TRAP activity from the cell culture supernatant. (F) CTSK activity from the cell lysates. (G) MMP9 ELISA from the cell culture supernatant. (H) Western blotting for CTSK, MMP9, and ACP5/TRAP in BMMs treated with BafA1 (10 nM), CQ (50 nM), or ZOL (1 μM). Graphs show the mean ± SD. *p < 0.05, **p < 0.005, ***p < 0.0005, ****p < 0.0001. ns, not significant. Two-way ANOVA with Sidak multiple comparison test (E, F, G).

### ZOL impairs bone resorptive functions of osteoclasts

In clinical settings, BPs are typically present in a bound form on the bone, and free-form BPs are only available when fully differentiated mature osteoclasts begin resorbing onto the bone surfaces [24,25]. To recapitulate this *in vivo* setting, we pre-treated dentin slices with ZOL, removed unbound ZOL by extensive washing, and subsequently induced osteoclast differentiation of BMMs on the ZOL-treated dentin slices (Figure 3A). On these dentin slices, osteoclast differentiation remained unaffected on both PBS-treated and ZOL-treated dentin slices (Figure 3B,C). In contrast, the bone resorptive function was severely impaired in ZOL-treated dentin slices (Figure 3D,E). To further confirm this, we differentiated osteoclasts on PBS- or ZOL-treated dentin and stained F-actin with phalloidin. Phalloidin imaging showed intact F-actin sealing rings on both dentins (Figure S2A), indicating preserved cytoskeletal polarization. However, pit depth from confocal Z-stacks was reduced on ZOL-pretreated dentin (Figure 3F,G; Figure S2B), consistent with impaired secretory resorption despite normal actin-ring formation. Additionally, we collected supernatants from the dentin slice assay and found significantly decreased levels of the C-terminal telopeptide of type I collagen (CTX-I) and MMP9 in the presence of ZOL (Figure 3H,I). Taken together, these findings indicate that BPs at non-apoptotic doses impair the bone resorptive functions of osteoclasts.

**Figure 3.**
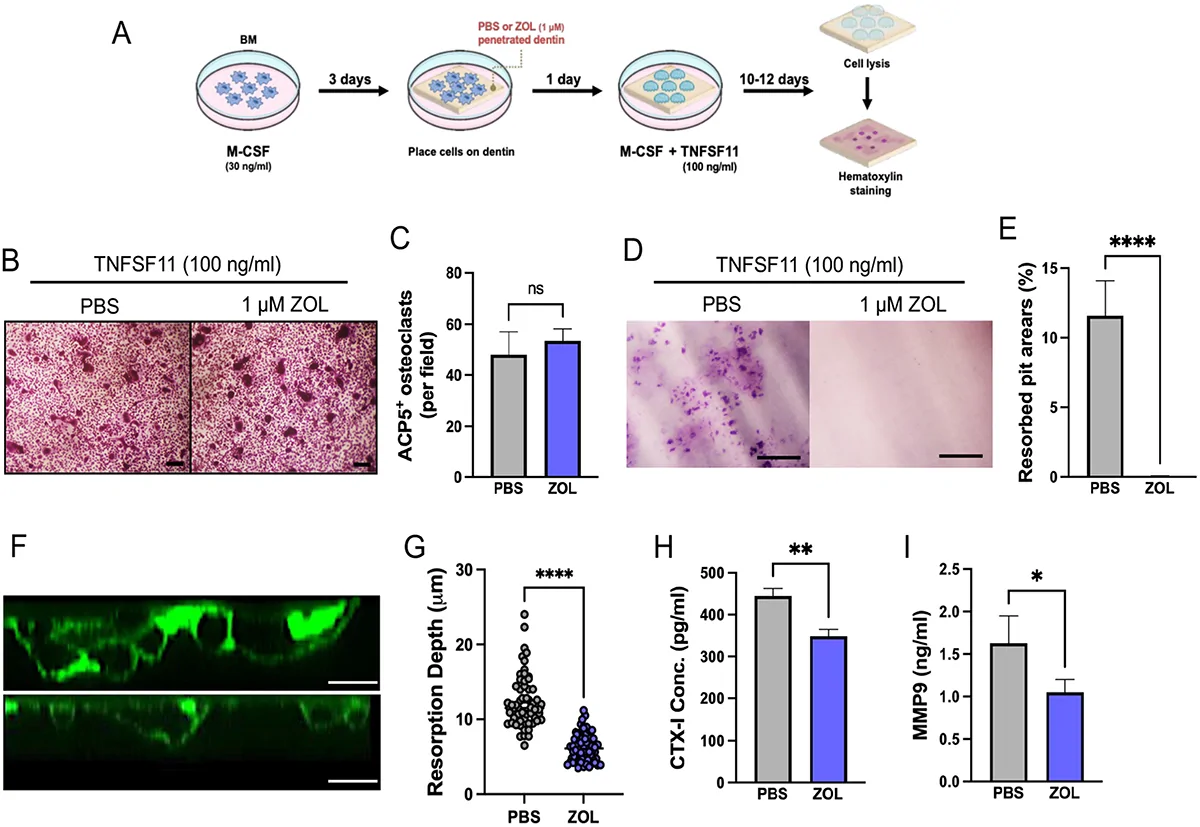
ZOL impairs bone resorptive functions of osteoclasts. (A) Schematic diagram of the dentin slice assay. (B) ACP5/TRAP staining of BMMs allowed to undergo osteoclast differentiation on the ZOL-treated dentin slice for 10 days. Scale bars: 200 μm. (C) Quantification of ACP5/TRAP-positive cells. (D) Hematoxylin staining in the dentin slice assay after removing osteoclasts. Scale bars: 100 μm. (E) Quantification of hematoxylin-stained bone resorptive pits. (F) Osteoclasts were differentiated on PBS- or ZOL-pretreated dentin, and cells were stained with F-actin with phalloidin. The resorbed pit depth on the dentin slice was visualized in a z-stack by confocal microscopy. Scale bars: 10 μm. (G) the image was reconstructed and quantified by Imaris software. CTX-I amount (H) and MMP9 ELISA (I) were measured from the supernatants obtained from the dentin slice assay (day 12). Graphs show the mean ± SD. *p < 0.05, **p < 0.005, ****p < 0.0001. ns, not significant. Unpaired t-test.

### ZOL inhibits RAB7 prenylation and suppresses osteoclast differentiation

BPs physically bind to and inhibit the function of farnesyl pyrophosphate synthase (FPPS), a key branch-point enzyme in the mevalonate pathway, which leads to target protein prenylation, including farnesylation and geranylgeranylation [26] (Figure 4A). Geranylgeranylation is an important factor in osteoclast formation, function, and survival [27]. To examine whether protein prenylation is an important step in BP-mediated inhibition of osteoclasts’ function, we used GGTI-2133, a geranylgeranyl transferase inhibitor, and NE10790, a specific RAB GGtase II inhibitor, and found that both NE10790 and GGTI-2133 suppressed osteoclast differentiation in a dose-dependent manner (Figure 4B,C). When the subunits of Ggtase-I and Ggtase-II were inhibited by *Pggt1b* (protein geranylgeranyltransferase type I subunit beta) siRNA (*Pggt1b*-si) and *Rabggtb* (Rab geranylgeranyltransferase subunit beta) siRNA (*Rabggtb*-si), respectively (Figure 4D,E), osteoclast formation was suppressed by *Rabggtb* siRNA but not *Pggt1b* siRNA (Figure 4F,G), suggesting that protein geranylation of RAB family members is important in osteoclast differentiation. Further analysis using proteomics in ZOL-treated BMMs revealed downregulation of small GTPase-mediated signal transduction (Figure S3). Among RAB family members, RAB7 is a small GTPase that localizes to the ruffle border [28,29] and plays a vital role in osteoclast function by transporting from early to late endosomes or from late endosomes to lysosomes [30,31]. Because the prenylation of small GTPases determines the downstream signaling pathway [32,33], we next examined whether RAB7 prenylation was affected by BPs. Upon CSF1/M-CSF (colony stimulating factor 1) and TNFSF11/RANKL stimulation, RAB7 underwent prenylation (Figure 4H,P: prenylated Rab7) in a time-dependent manner in BMMs. However, in the presence of ZOL, RAB7 prenylation did not occur (Figure 4H,U: unprenylated Rab7). RAB7 prenylation was found to be unaffected in the presence of autophagy inhibitors BafA1 or CQ, suggesting that RAB7 prenylation is specific to ZOL (Figure 4I). In contrast, RAB7 prenylation inhibition by ZOL occurred in a dose-dependent manner, which was also reflected in the accumulation of ATG-related SQSTM1/p62 protein and the diminution of matured CTSK (Figure 4J). N-BPs have been reported to influence prenylation of multiple small GTPases; but prenylation of other small GTPases, such as RHOA or RAB5, was not observed (Figure S4). These data indicate that the inhibition of RAB7 prenylation is associated with the maturation of secretory proteins in ZOL-treated osteoclasts.

**Figure 4.**
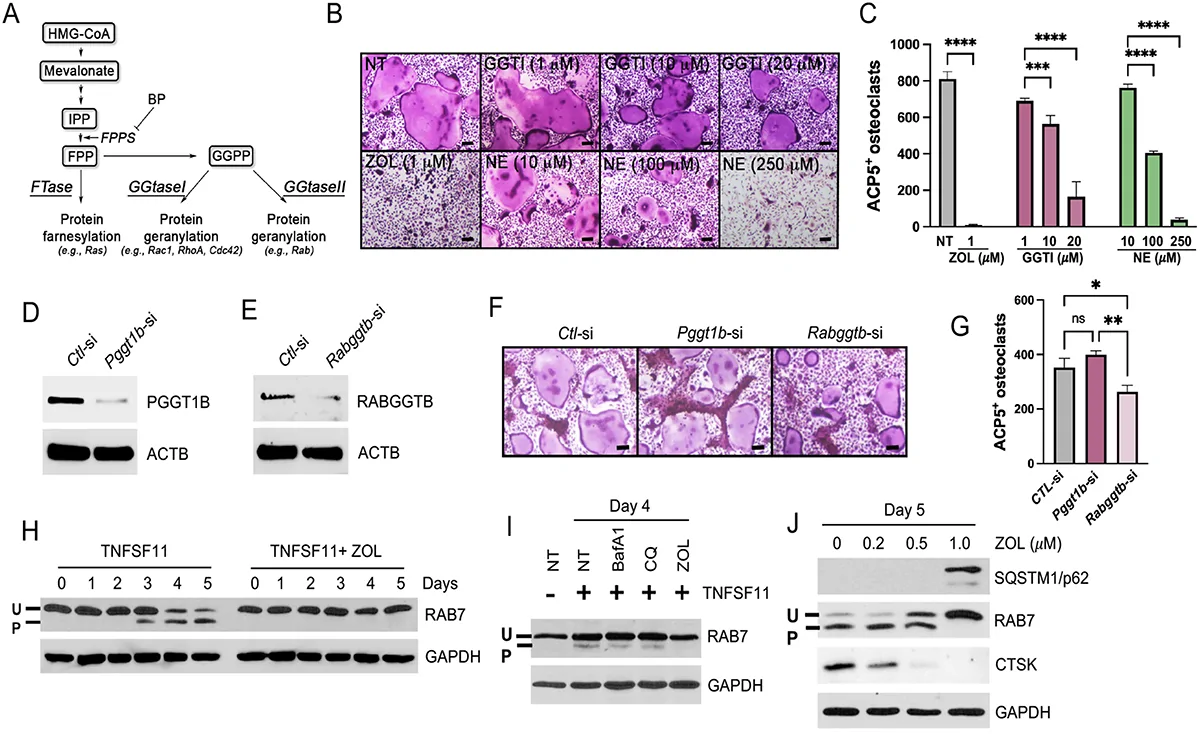
ZOL inhibits Rab7 prenylation required for osteoclast differentiation. (A) Schematic diagram of the mevalonate pathway. (B) ACP5/TRAP staining of osteoclasts treated with ZOL, GGTI, or NE at the indicated doses. Scale bars: 200 μm. (C) Quantification of ACP5/TRAP-positive cells. Western blotting for PGGT1B (D) and RABGGTB (E) after knockdown of these genes using siRNA technology. (F) ACP5/TRAP staining of BMMs transfected with siRnas targeting *Pggt1b* and *Rabggtb*. Scale bars: 200 μm. (G) Quantification of ACP5/TRAP-positive cells. (H) Western blotting for RAB7 and GAPDH during osteoclast differentiation in the absence or presence of 1 μM ZOL. U: unprenylated RAB7, P: prenylated RAB7 (I) Western blotting for RAB7 and GAPDH in the presence of BafA1 (10 nM), CQ (50 nM), or ZOL (1 μM) during osteoclast differentiation. (J) Western blotting for SQSTM/p62, RAB7, CTSK, and GAPDH during osteoclast differentiation with different doses of ZOL. Graphs show the mean ± SD. *p < 0.05, **p < 0.005, ***p < 0.0005, ****p < 0.0001. ns, not significant. One-way ANOVA with Tukey’s multiple comparison test (C and G).

### rab7 cKO mice exhibit osteopetrotic phenotypes in vivo

To demonstrate the functional role of RAB7 in osteoclasts *in vivo*, we generated osteoclast-specific conditional knockout mice: *rab7*^fl/fl^; *Ctsk*^*Cre/+*^ (*rab7* cKO) mice and littermate *Rab*7^f/f^; *Ctsk*^*+/+*^ controls (*Rab7* WT) mice (Figure S5A). Because *in vivo* effects of the bound-form of BPs do not inhibit osteoclast differentiation but osteoclast function only when matured osteoclasts start up-taking the free-form of BPs upon bone resorption, we strategically used Cre mice driven by the *Ctsk* promoter to knockout *Rab7* gene, which allows for deleting *Rab7 gene* only when osteoclasts become fully differentiated. Because the genotype effect was concordant in both sexes, we pooled females and males for Figure 5; the sex-stratified results (females and males shown separately) were provided in Figure S5. At 2 months, *rab7* cKO mice had lower body weight and shorter femora than *Rab7* WT mice (Figure 5A-D; sex-stratified in Figure S5B and C). Spleen weight was comparable between genotypes, whereas spleen/body weight was higher in females owing to reduced body mass (Figure S5D and E). µCT of the distal femur revealed a consistent osteopetrotic architecture in cKO mice (Figure 5E): increased BV/TV (bone volume/tissue volume) (Figure 5F; Figure S5F), more trabeculae (Figure 5G; Figure S5G), and lower separation (Figure 5H; Figure S5H). A modest increase in cortical thickness was also observed (Figure 5I), but there is no significant difference in the male group (Figure S5I). The vertebra showed the same architectural trend (Figure S6A-D). When the serum bone turnover markers were measured, the resorption marker CTX-I was reduced, whereas the formation marker procollagen type I N-terminal propeptide (P1NP) was increased in *rab7* cKO mice (Figure 5J,K). Further correlation analysis with individual mice showed that P1NP and CTX-I levels are inversely correlated (Figure S5N and O), indicating that the overall bone formation increased while bone resorption decreased. Histological examination of the femurs showed fewer marrow adipocytes in *rab7* cKO mice for both sexes (Figure S6K and L), results of which are consistent with reduced marrow space in osteopetrotic bone. At 6 months, *rab7* cKO mice remained high in BT/TV; however, the differences in body weight and spleen/body weight had become insignificant, while femur length remained reduced (Figure S6E-J), suggesting that osteoclast-specific deletion of *Rab7* did not affect the overall health of the mice. Histomorphometric analysis revealed that both osteoblast number (N. Ob) and osteoblast number per bone perimeter (N.Ob/B.Pm) were not affected in both groups of mice (Figure 5L-N; Figure S5J and K), while total osteoclast number (N.Oc) was increased with unchanged osteoclast number per bone perimeter (N.Oc/B.Pm) in *rab7* cKO mice (Figure 5O-Q; Figure S5L and M), suggesting that osteoclast-specific deletion of *Rab7* does not affect osteoclast differentiation *in vivo*. Taken together, *rab7* deletion produces a reproducible osteopetrotic phenotype characterized by greater trabecular bone mass.

**Figure 5.**
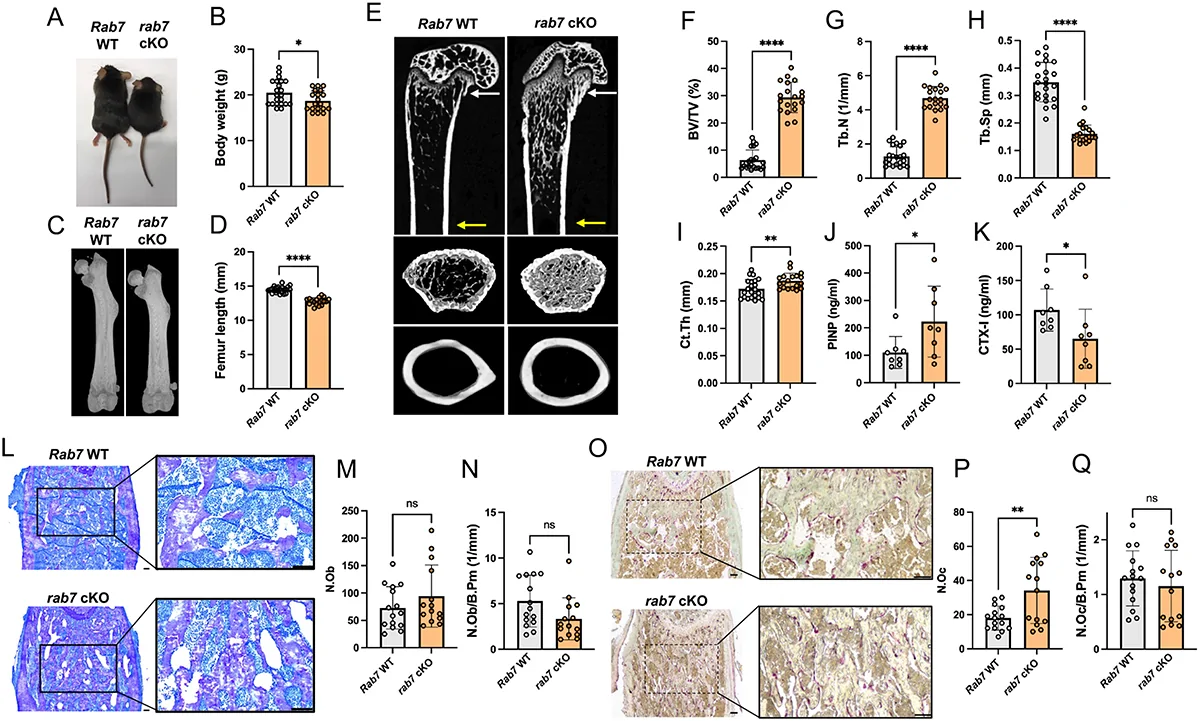
*rab7* cKO mice exhibit osteopetrotic phenotypes in vivo. (A) Representative images of male *Rab7* WT and *rab7* cKO mice at 8 weeks of age. (B) Body weights at the eighth week (*Rab7* WT: *n* = 22, *rab7* cKO: *n* = 19). (C) Representative μCT image of the femur in *Rab7* WT and *rab7* cKO mice (male). (D) Femur lengths were measured at the eighth week (*Rab7* WT: *n* = 22, *rab7* cKO: *n* = 19). (E) Representative μCT image of femurs from *Rab7* WT and *rab7* cKO mice (male). Top: longitudinal cross-sections of the distal femur. White arrows mark the plane of the distal metaphyseal trabecular cross-sections shown in the middle row; yellow arrows mark the plane of the mid-diaphyseal cortical cross-sections shown in the bottom row. (F–I) Quantification at 8 weeks: bone volume/tissue volume (BV/TV), trabecular number (Tb.N), trabecular separation (Tb.Sp), and cortical thickness (Ct.Th) (*Rab7* WT: *n* = 22, *rab7* cKO: *n* = 19). (J and K) Serum was collected from *Rab7* WT and *rab7* cKO mice (sex-pooled; *n* = 8 per group, males = 3, females = 5). (J) Procollagen type I N-terminal propeptide (P1NP, formation marker). (K) C-terminal telopeptide of type I collagen (CTX-I, resorption marker). (L) Toluidine blue staining of the femur (female). Scale bars: 100 μm. Quantification of osteoblast number (N.Ob) (M) and osteoblast number per bone perimeter (N.Ob/B.Pm) (N). (O) TRAP staining of the femur (female). Scale bars: 100 μm. Quantification of osteoclast number (N.Oc) (P) and osteoclast number per bone perimeter (N.Oc/B.Pm) (Q). Sex-pooled, *Rab7* WT: *n* = 15, *rab7* cKO: *n* = 14 (L-Q). Bars show pooled sexes with individual data points; sex-stratified datasets are in Figure S5. Graphs show the mean ± SEM. *p < 0.05, **p < 0.005, ***p < 0.0005 ****p < 0.0001. n.S., not significant. Unpaired t-test.

### RAB7 is required for osteoclasts’ bone resorptive functions

The *rab7* cKO mice exhibited osteopetrotic phenotype without affecting the number of osteoclasts. Therefore, we asked whether these mice have osteoclasts that are functionally impaired. To do so, BMMs were isolated from *rab7* cKO mice and subjected to osteoclast differentiation. Upon TNFSF11/RANKL stimulation, *Ctsk*-Cre expression increased while *Rab7* expression decreased (Figure 6A,B). When these cells were allowed to undergo differentiation, the number of osteoclasts was not affected, indicating that *Ctsk*-driven Cre expression did not affect *Rab7* during osteoclast differentiation (Figure 6C,D). However, the dentin slice assay showed significantly suppressed bone-resorptive function of osteoclasts derived from *rab7* cKO mice (Figure 6E,F). The western blotting from the cells collected from the dentin slice confirmed that RAB7 and matured CTSK expression were significantly diminished (Figure 6G). To test whether RAB7 is required for autophagy-dependent secretion, BMMs were transfected with *Rab7* siRNA (*Rab7-*si) or *control* siRNA (*Ctl-*si) under CSF1/M-CSF and then differentiated to osteoclasts with TNFSF11/RANKL. RAB7 protein was efficiently depleted by siRab7 (Figure 6H). At day 3, cells were treated with or without BafA1 and MAP1LC3/LC3 was immunoblotted to quantify autophagic flux. The BafA1-induced increase in MAP1LC3B-II was significantly smaller in *Rab7* siRNA than in *control* siRNA osteoclasts (Figure 6I), indicating reduced autophagic flux upon *Rab7* knockdown (Figure 6J). Functionally, ACP5/TRAP activity in conditioned medium was decreased in *Rab7* siRNA osteoclasts (Figure 6K), consistent with impaired autophagolysosomal secretion. Together, these data suggest that RAB7 has a significant role in mediating the bone resorptive functions, but not differentiation, of osteoclasts.

**Figure 6.**
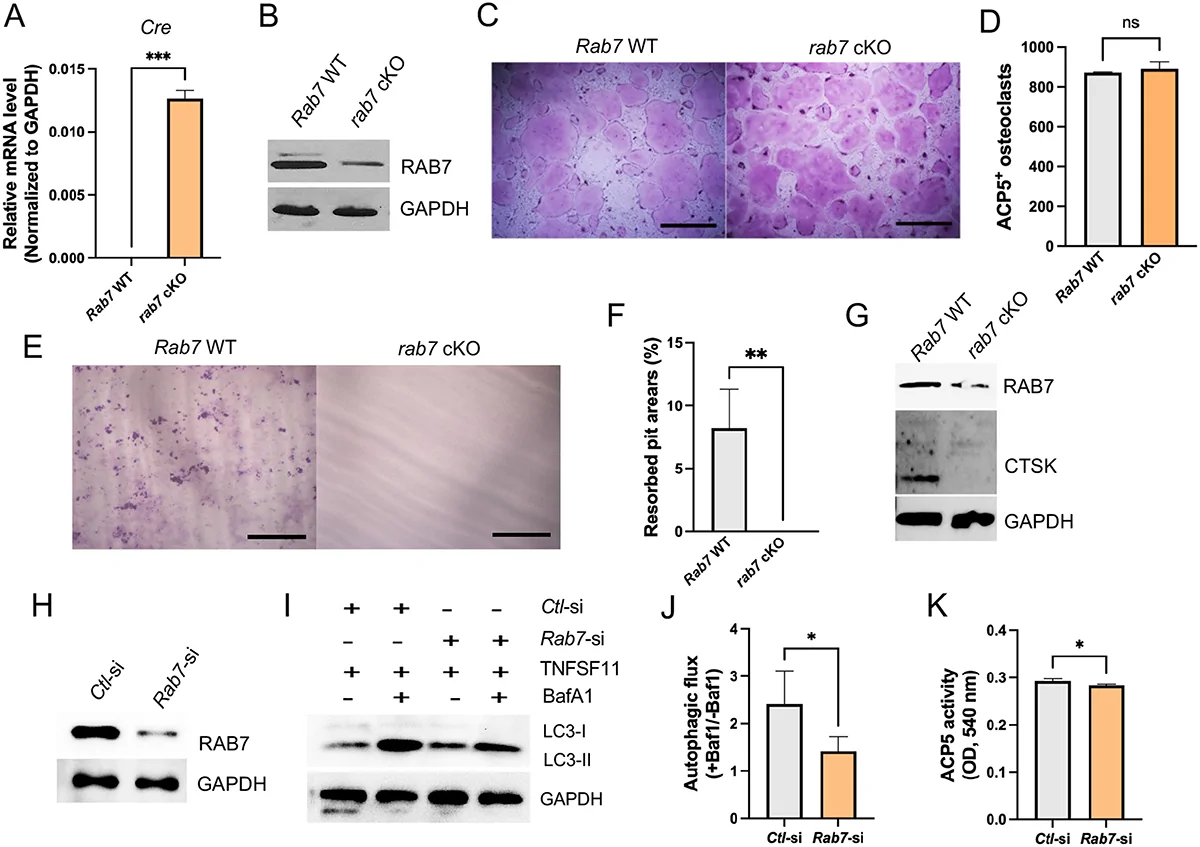
*Rab7*-deficient osteoclasts exhibit defective bone resorptive functions. (A) qRT-PCR for Cre in BMMs isolated from *Rab7* WT or *rab* cKO mice (8-week-old female; one mouse from each group) after inducing osteoclast differentiation. Cells were harvested at day 4. (B) Western blotting of RAB7 and GAPDH. (C) ACP5/TRAP staining of BMMs isolated from *Rab7* WT or *rab* cKO mice. Scale bars: 500 μm. (D) Quantification of ACP5/TRAP-positive cells. (E) Hematoxylin staining in the dentin slice assay using BMMs isolated from *Rab7* WT or *rab* cKO mice. (F) Quantification of Hematoxylin-stained bone resorptive pits. Scale bars: 100 μm. (G) Western blotting of RAB7, CTSK, and GAPDH from the cells on the dentin slices. (H-K) BMMs (8-week-old WT female; one mouse) transfected with *control* siRNA (*Ctl-*si) or *Rab7* siRNA (*Rab7*-si) were differentiated (CSF1/M-CSF and TNFSF11/RANKL, 3d). (H) RAB7 knockdown was confirmed by western blot. (I) Western blot for MAP1LC3/LC3 and GAPDH from the same cultures treated with or without BafA1. (J) Quantification of autophagy flux (*n* = biological triplicates). Graphs show the mean ± SEM. (K) ACP5/TRAP activity from the cell culture supernatant. Graphs show the mean ± SD (A, D, and K). *p < 0.05, **p < 0.005, ***p < 0.0005. ns, not significant. Unpaired t-test.

## Discussion

In the present study, we examined the effects of BPs on osteoclasts at non-apoptotic doses and found that BPs did not induce apoptosis but suppressed autophagy in osteoclasts. We further found that BPs inhibit maturation and secretion of secretory proteins such as CTSK, ACP5/TRAP, or MMP9 that are responsible for bone resorption, and that such inhibition occurs in a Rab7-dependent manner. This study identifies BPs as putative autophagy inhibitors that suppress osteoclast function by blocking the autophagy pathway.

Although apoptosis remains one of the most well-known cellular responses in osteoclasts by BPs [2], the precise molecular mechanisms by which BPs suppress the bone resorptive functions of osteoclasts remain controversial. Many studies have demonstrated the apoptotic effects of BPs on osteoclasts in *in vitro* and *in vivo* studies, but close examinations reveal that they used high doses of BPs [2,34,35]. On the other hand, at the lowest effective dose, apoptosis was not required to inhibit bone resorption by BPs [36], suggesting that BPs may inhibit bone resorption independent of inducing apoptosis. Indeed, in line with the latter observations, we found that low doses of ZOL were sufficient to inhibit bone resorptive functions without causing apoptosis, and this is primarily due to suppressing the autophagy pathway in the osteoclasts.

Previous reports have suggested that BPs induce autophagy in various cells as determined by increased MAP1LC3-II levels [37–39]. Consistent with these observations, our study showed that ZOL also induced MAP1LC3-II expression in osteoclasts. However, an increase in MAP1LC3-II levels indicates either enhanced autophagosome formation or impaired autophagic flux, resulting in the accumulation of undegraded autophagosomes and MAP1LC3-II [40]. When autophagy flux was determined, there was a significant increase in MAP1LC3-II level, indicating that ZOL caused accumulation of MAP1LC3-II by blocking the autophagic flux. Similarly, time-dependent treatment of ZOL delayed the degradation of autophagy-related proteins that become degraded via the autophagic pathway. Autophagy inhibitors, such as CQ and BafA1, interfere with lysosomal function during the autophagy process, resulting in the accumulation of autophagy-related proteins, indicating that the increased levels of MAP1LC3-II protein caused by ZOL in osteoclasts are due to the blocking of autophagy and not the induction of autophagy.

An increased lines of evidence suggest that autophagy is involved in the secretion of extracellularly released proteins [14–16]. A recent study showed that Beclin1, an autophagy protein that is indispensable in autophagy initiation, plays a critical role in osteoclasts [12]. In this study, we found that during osteoclast differentiation, ZOL treatment efficiently inhibited the maturation of CTSK, ACP5/TRAP, and MMP9 by blocking conversion of their pro-forms into cleaved forms [4,5]. The results from the dentin slice assay also showed a significant decrease in the levels of CTX-I and MMP9 in the supernatants of ZOL-treated dentin. Similar to the effect of ZOL treatment, autophagy inhibitors inhibited the maturation of secretory proteins, suggesting that autophagy is a critical pathway mediating the processing of secretory proteins in osteoclasts.

BPs spontaneously bind to calcium-enriched bone surfaces [24]. Bone-bound BPs remain on bone surfaces for years with an expected half-life of over 10 years. During active resorption, bone-bound drug is exposed within the resorption lacuna and taken up by osteoclasts [41–43]. In our dentin-pretreated model, osteoclasts differentiated normally on ZOL-bound dentin (intact actin rings) because no free-form of ZOL was available to interfere with osteoclast differentiation. However, these osteoclasts were unable to complete bone resorption. It is likely that, once resorption is commenced, bound-form of ZOL was taken up by the cells and blocked maturation of secretory proteins important for bone resorption by inhibiting secretory autophagic pathway, leading to shallower pits and reduced CTX-I/MMP9 release. Thus, the data are consistent with osteoclast-targeted bioavailability of bone-bound ZOL and explain why differentiation appears intact while resorptive function is impaired.

RAB7 is a small GTPase required for normal autophagy [44,45]. RAB7 is prenylated by GGtase II in the mevalonate pathway [46,47], and prenylated RAB7 plays a critical role in autophagy, whereas unprenylated RAB7 inhibits autophagosome turnover [33,48]. During osteoclast differentiation, RAB7 was prenylated in a time-dependent manner. After ZOL treatment, RAB7 prenylation was completely abolished, and the inhibition of RAB7 prenylation and CTSK maturation occurred in a dose-dependent manner. These data suggest that inhibition of prenylation of RAB7 is a critical step in the inhibition of osteoclast function mediated by ZOL.

RAB7 plays a critical role in osteoclast polarization and bone resorption [30], but its functional importance in *in vivo* has never been reported previously. Our study showed that osteoclast-specific *rab7* deletion produced a robust osteopetrotic bone phenotype by inhibiting bone-resorptive functions of osteoclasts. Interestingly, despite the increase in bone mass, calcein double labeling revealed significant decreases in mineral apposition rate (MAR), bone formation rate per bone surface (BFR/BS), and mineralizing surface per bone surface (MS/BS) in *rab7* cKO mice in both femur and vertebrae (Figure S7). Diminished MAR, BFR/BS, and MS/BS in mice with osteopetrotic phenotypes has been previously reported in mice in which osteoclast differentiation was completely abolished by targeting TNFSF11/RANKL, a key inducer of osteoclast differentiation [49–51]. It is then likely that *rab7* deletion in osteoclasts is potent, comparable to TNFSF11/RANKL inhibition, in suppressing bone-resorptive functions of osteoclasts.

Our data highlight RAB7 as a key target of ZOL; however, other RAB GTPases or trafficking pathways may also be affected. However, ZOL had no effects of prenylation of other RAB GTPases, at least on RAB5 or RHOA (Figure S4), suggesting that there is specificity of ZOL effect in osteoclasts. Although RAB7 shows promise as a therapeutic target, further validation is needed to determine whether modulating its prenylation or autophagic activity is effective and safe *in vivo*.

In summary, we have demonstrated that ZOL blocks autophagy and impairs maturation of bone-resorbing secretory proteins such as CTSK, MMP9, or ACP5/TRAP by inhibition of RAB7 prenylation, leading to incompletion of bone resorption by osteoclasts (Figure 7). These findings uncover a novel, apoptosis-independent mechanism of ZOL action and establish RAB7-mediated secretory autophagy as essential for osteoclast function. Clinically, this suggests ZOL disrupts vesicular trafficking required for enzyme secretion, offering a new perspective on its anti-resorptive effects. Targeting autophagy, especially via RAB7 or related GTPases, may provide a more targeted strategy to treat bone diseases such as osteoporosis and metastases, with improved specificity and fewer side effects.

**Figure 7.**
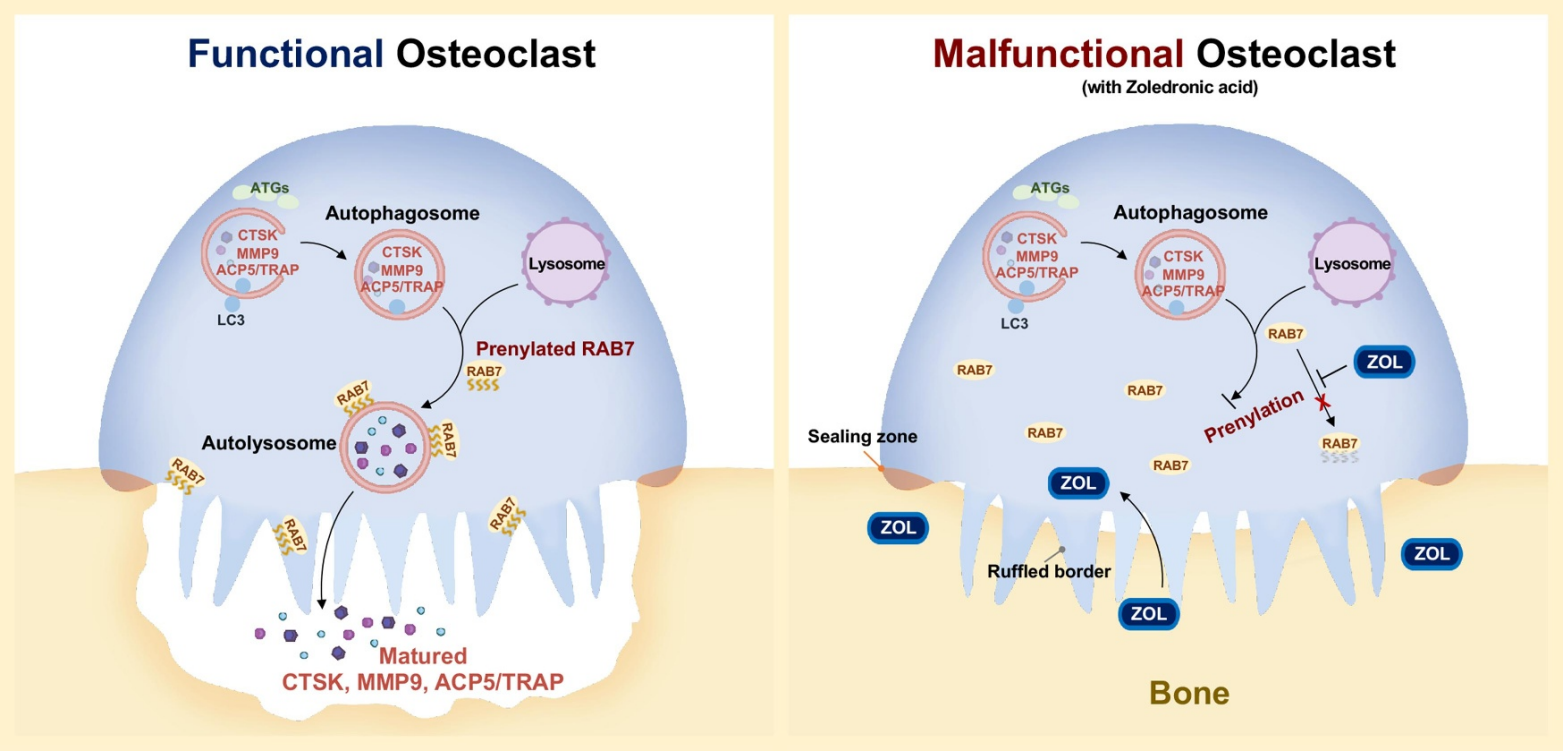
Graphic summary of the study. Functional osteoclast (left): in the absence of ZOL, an autophagosome engulfs bone-resorbing secretory proteins (e.g., CTSK, MMP9, and ACP5/TRAP), merges with a lysosome in a RAB7-prenylation-dependent manner. The autolysosome releases matured CTSK, MMP9, and ACP5/TRAP into the pits to resorb bone surfaces. Malfunctional osteoclast (right): in the presence of ZOL, the drug first binds to the bone surface. During active resorption, osteoclasts take up bone-bound ZOL, which prevents RAB7 prenylation. Without prenylated RAB7, autophagosomes containing bone-resorbing secretory proteins (e.g., CTSK, MMP9, and ACP5/TRAP) cannot merge with the lysosome, leading to defective bone resorption (malfunctional osteoclasts).

## Materials and methods

### Reagents and antibodies

The following reagents were purchased: Zoledronic Acid (ZOL; LKT laboratories, Z5744), bafilomycin A_1_ (BafA1; Millipore-Sigma, B1793), chloroquine (CQ; Millipore-Sigma, C6628), GGTI-2133 (Millipore-Sigma, G5294), and 3-PEHPC (known as NE 10,790; MedChemExpress, HY-16011). The antibodies used for the western blot analysis were: CASP3 (Cell Signaling Technology, 9661), GAPDH (Santa Cruz Biotechnology, sc-25778), CTSK (Abcam, ab66237), MMP9 (Abcam, ab19016), LC3B (Cell Signaling Technology, 2775), SQSTM1/p62 (Cell Signaling Technology, 5114), BECN1 (Cell Signaling Technology, 3495), ATG4B (MBL, M134-3), ATG7 (MBL, PM039B), ACTB/β-ACTIN (Santa Cruz Biotechnology, sc-47778), LAMP1 (Abcam, ab25630), RAB7 (Cell Signaling Technology, 9367), RABGGTB (Abcam, ab187717), and PGGT1B (Abcam, ab122122). ACP5/TRAP antibody that recognizes the monomeric 5a (loop epitope) was obtained from Dr. Goran Andersson, Karolinska University Hospital, Sweden) [22]. Secondary antibodies: HRP-conjugated goat anti-rabbit IgG (H+L) (Jackson ImmunoResearch, 111–035-003) and HRP-conjugated goat anti-mouse IgG (H+L) (Jackson ImmunoResearch, 115–035-003).

### Mouse model

*Rab7*^*f/f*^ (loxP sites flanking exon 1 of *Rab7*) mice were purchased from the Jackson Laboratory (021589), and *Ctsk*^*Cre/+*^ mice were kindly provided by Dr. Nakamura Takashi (Keio University, Japan) [52]. These mice were crossbred to generate *Rab7*^*f/f*^;* Ctsk*^*+/+*^ mice (*Rab7* WT) mice and their littermates, *rab7*^*f/f*^;* Ctsk*^*Cre/+*^ (*rab7* cKO) mice. Genotyping primer sequences are listed in Table S1; representative gels are shown in Figure. S5A. The mice were maintained under specific pathogen-free (SPF) conditions. For animal study, all animal experiment protocols were approved (ARC protocol #2012–033 and #2011–062) and conducted according to the Animal Research Committee and the UCLA Institutional Animal Care and Use Committee (IACUC).

### Micro-computed tomography (µCT) scan and three-dimensional volumetric analysis

After euthanasia, femora were carefully dissected free of soft tissues with fine scissors and forceps, avoiding damage to cortical surfaces. Bones were fixed in 4% paraformaldehyde (PFA) in PBS (Millipore-Sigma, 158,127) for 24 h at 4°C with gentle rocking. Following fixation, specimens were rinsed three times in PBS (5 min each) to remove residual fixative and then transferred to 70% ethanol for storage at 4°C until μCT scanning.

Areas of interest on the femur were subjected to μCT scanning (SkyScan1275, Bruker, Kontich, Belgium) at 60 kVp and 166 μA using a voxel size of 10 μm^3^ and a 0.5 mm Aluminum filter with an integration time of 200 ms using a cylindrical tube (FOV/Diameter: 20.48 mm). Two-dimensional slices from each femur were combined using the SkyScan NRecon software to form a three-dimensional reconstruction. Trabecular architecture was quantified in the distal femoral metaphysis. The ROI was defined as 360 consecutive slices immediately proximal to the distal growth plate (secondary spongiosa), excluding the cortical shell. Cortical parameters were measured in a mid-diaphyseal ROI centered at 50% of femur length (fixed axial span). The image analysis was performed using the DataViewer and CTAn software. Trabecular and cortical microarchitecture were evaluated in accordance with ASBMR guidelines [53].

### Cells and cell culture

RAW 264.7 cells were purchased from ATCC (TIB-71) and cultured in α-MEM (Gibco, 12,571,063) containing 10% fetal bovine serum (FBS; Gibco, 10,437,028). Bone marrow macrophages (BMMs) were obtained from tibiae and femur of adult C57/BL6 mice (4–6 weeks old), *Rab7*^*f/f*^;* Ctsk*^*+/+*^, or *rab7*^*f/f*^;* Ctsk*^*Cre/+*^ mice and cultured in α-MEM containing 10% FBS in the presence of 30 ng/ml recombinant mouse CSF1/M-CSF (R&D Systems, 416ML010CF). Bone marrow cells were plated at 3 × 10^5^ cells/mL in 60-mm dishes with CSF1/M-CSF (30 ng/mL) for 3 days. Non-adherent cells were discarded by medium change, and the adherent cells were designated as bone-marrow macrophages (BMMs). For osteoclast differentiation, BMMs were further cultured with 100 ng/ml recombinant mouse TNFSF11/RANKL (R&D Systems, 462-TR) in the presence of 30 ng/ml CSF1/M-CSF.

Osteoclast formation was assessed by ACP5/TRAP staining (Leukocyte Acid Phosphatase Kit; Millipore-Sigma, 387A) following the manufacturer’s instructions. Cells were cultured in 48-well plates. Per well, four non-overlapping fields were imaged at a fixed magnification. ACP5/TRAP^+^ multinucleated cells (≥3 nuclei) were counted per field; field counts were summed to a per-well value. *n* = 3 wells per condition were analyzed per experiment.

### Vectors and vector construction

To construct the pLPCX-RFP-GFP-LC3 plasmid, a DNA fragment encoding RFP-EGFP-LC3 was cloned into the pLPCX retroviral expression vector. The RFP-EGFP-LC3-encoding cDNA was inserted into the vector at the XhoI and EcoRI restriction sites. The RFP-EGFP-LC3 plasmid was generously provided by Prof. Jeon-Soo Shin (Yonsei University, Seoul, Korea).

### Virus production and transduction of cells

Retroviral vectors were transfected into GP2-293 universal packaging cells (TAKARA, 631,458) along with the pVSV-G envelope plasmid using Lipofectamine 2000 (Life Technologies, 11,668,027). Forty-eight h post-transfection, the virus-containing supernatant was collected and concentrated by ultracentrifugation. The resulting viral pellet was resuspended in α-MEM and either used immediately for infection or stored at −80°C for future use. Target cells were infected with the retrovirus in the presence of 6 μg/mL polybrene for 3 h.

### Western blotting

Cells were washed twice with PBS before treatment with ice-cold lysis buffer (20 mM Tris-HCl, pH 7.4, 150 mM NaCl, 5 mM EDTA, 1% Triton X-100 [Millipore-Sigma, T8787], and phosphatase inhibitor cocktail II [Millipore-Sigma, P5726]). Then, the cells were scraped and incubated on ice for 10 min. Cell debris was separated by centrifugation at 20,000 g at 4°C for 20 min, and the supernatant was collected for western blot analysis after 12 or 15% SDS-PAGE. After electrophoresis, proteins were transferred to a nitrocellulose blotting membrane (GE Healthcare Life Science, 10,600,016), which was subsequently blocked with 5% nonfat milk for 1 h at room temperature. Then, the membrane was incubated with primary antibodies and probed with the respective secondary antibodies conjugated with HRP. The signals were obtained using ChemiDoc Touch Image System (Bio-Rad).

### siRNA transfection

BMMs were transfected with interference RNA for control (*control* siRNA; Santa Cruz Biotechnology, sc37007), *Pggt1b* (*Pggt1b* siRNA; Santa Cruz Biotechnology, sc-77357), or *Rabggtb* (*Rabggtb* siRNA; Santa Cruz Biotechnology, sc-45438) individually with RNAiMAX (Life Technologies, 13,778,075) according to the manufacturer’s instructions. Two days after transfection, cells were cultured for 5 days in the presence of CSF1/M-CSF and TNFSF11/RANKL for osteoclast differentiation. For the *Rab7* knockdown flux experiments, BMMs were transfected with interference RNA for control (*control* siRNA; Santa Cruz Biotechnology, sc37007) or *Rab7* (*Rab7* siRNA; Santa Cruz Biotechnology, sc-270071) individually with RNAiMAX under CSF1/M-CSF, then induced with CSF1/M-CSF and TNFSF11/RANKL for 3 days.

### Real-time quantitative RT-PCR (qRT-PCR)

qRT-PCR was performed as described previously [54]. Briefly, the cells were harvested with 1 mL Trizol Reagent (Invitrogen, 15,596,026) per plate. mRNA was extracted with PureLink RNA kit (Invitrogen, 12,183,025), and cDNA was synthesized with PCR (SuperScript III Reverse Transcriptase; Invitrogen, 18,080,085). Real-time PCR was done using PowerUp SYBR Green Master Mix (Invitrogen, A25742). A total of 45 cycles were executed, and the second derivative of the Cq value determination method was used to compare fold differences.

### Confocal microscopy

BMMs were cultured in 4-well chambers (Millipore-Sigma, 154,526) in α-MEM supplemented with 10% FBS. Cells were infected with retroviruses expressing pLPCX-RFP-GFP-LC3 and treated with CSF1/M-CSF and TNFSF11/RANKL to induce differentiation into osteoclasts over 3 to 4 days. To assess autophagy, cells were treated with 10 nM BafA1 or 1 μM Zol for 16 h, then fixed with 4% formaldehyde. Fixed cells were mounted using Vectashield Mounting Medium with DAPI (Vector Laboratories, H-1200) and visualized using a FluoView FV10i confocal microscope (Olympus). For quantification, cells were counted with yellow puncta (GFP and RFP) and red-only puncta (RFP), and a GFP:RFP puncta ratio was calculated as a proxy for flux. For each condition, 40–50 cells per well were analyzed in *n* = 3 wells, and cell values were averaged to yield one per-well mean. Punta quantification was performed using Fluoview software (Olympus) and ImageJ.

For Z-stacks, osteoclasts were cultured on dentin, treated with PBS or ZOL, fixed, and stained with phalloidin (Millipore-Sigma, P5282) to label F-actin and DAPI. Samples were imaged by laser-scanning confocal microscopy, acquiring Z-stacks at 1-µm intervals. Resorption pit depth was quantified from orthogonal (XZ/YZ) reconstructions in Imaris software 10. 2. 0 (Andor Technology).

### CTSK and ACP5/TRAP activity and ELISA

To measure CTSK activity, ACP5/TRAP activity, and the level of MMP9 from cell culture supernatant during osteoclastogenesis, BMMs were differentiated on tissue-culture plastic (48-well) in CSF1/M-CSF and TNFSF11/RANKL. Cell culture supernatant was collected at days 0, 3, 4, and 5. Assays were performed per manufacturer’s instructions: CTSK (cathepsin K) activity (CTSK activity assay kit; Abcam, ab65303), Leukocyte Acid Phosphatase Kit (ACP5/TRAP activity; Millipore-Sigma, 387A), and MMP9 activity assay (QuickZyme Biosciences, QZBmmp9M).

For Dentin slice cultures, BMMs were differentiated on sterile dentin slices in CSF1/M-CSF and TNFSF11/RANKL. Supernatants were harvested at days 0, 10, and 12 for CTX-I (RatLaps® [CTX-I] EIA; IDS, AC-06F1) and MMP9 activity assay.

Serum concentrations of the CTX-I and procollagen type I N-terminal propeptide (P1NP) were quantified by ELISA using the RatLaps® (CTX-I) EIA and the Rat/Mouse P1NP EIA kit (IDS, AC-33F1), respectively, following the manufacturers’ instructions.

### Dentin slice assay

To measure the depth of resorption pits, BMMs (1 × 10^5^/ ml) isolated from *Rab7* WT or *rab* cKO mice were plated on dentin slices in 48-well culture plates (0.5 ml/well) and were treated with CSF1/M-CSF and TNFSF11/RANKL for 4 days. Then, dentin slices were moved to new 48-well culture plates, and cells were cultured for 8 days in the presence of CSF1/M-CSF and TNFSF11/RANKL. After cells were removed from dentin slices, the pits were stained with Mayer’s hematoxylin for 10 min and rubbed with cotton swabs to clear nonspecific staining. The resorbed pit was visualized under microscopy, and the percentage of the resorption pit area was quantified using ImageJ software. Resorption area (%) is defined as (pit area/total field-of-view area) × 100 for each field; field values were averaged per slice, and *n* = 3 dentins per condition were used for statistical comparison.

### Histochemical staining

The scanned tissues were decalcified with 5% EDTA (Millipore-Sigma, 798,681) and 4% sucrose (Millipore-Sigma, S0389) in phosphate-buffered saline, pH 7.4. Decalcification continued for 2 to 3 weeks at 4°C, and the decalcification solution was changed daily. Fully decalcified tissue samples were sent to the University of California, Los Angeles Translational Procurement Core Laboratory and processed for paraffin embedding. The embedded tissues were sectioned at 5-μm thickness. The sectioned slides were deparaffinized at 60°C and then rehydrated in ethanol with an increasing concentration of water. For ACP5/TRAP (acid phosphatase 5, tartrate resistant) staining, the rehydrated tissue slides were incubated for 1 h at 37°C with the ACP5/TRAP staining solution according to the manufacturer’s protocol (Leukocyte Acid Phosphatase Kit; Millipore-Sigma). The slides were counterstained with hematoxylin solution and mounted with ImmunoHistoMount (Millipore-Sigma, I1161). For H&E staining, the rehydrated tissue slides were stained with hematoxylin for 2.5 min, washed with water and 95% ethanol, and then stained with eosin for 1 min. The stained slides were dehydrated in 70%, 95%, and 100% ethanol, followed by xylene. The slides were mounted using a mounting medium (Cytoseal; Fisher Scientific, 23–244257). All images were taken through an Olympus microscope (model DP72; Olympus Corp.) at × 100 magnification.

### Bone histomorphometric analysis

Static and dynamic histomorphometry was carried out on *rab7*^*f/f*^;* Ctsk*^*Cre/+*^ (*rab7* cKO) mice and *Rab7*^*f/f*^;* Ctsk*^*+/+*^ (*Rab7* WT) controls at 8 weeks of age. Mice were injected with calcein twice, 20 mg/kg at an interval of 7 days, and the mice were sacrificed 2 days after the last injection. Femurs and Lumbar 3 (L3) were dissected, fixed in 70% ethanol, dehydrated, and embedded undecalcified in methyl methacrylate. Longitudinal sections, 5 µm thick, were cut on a Microm microtome (Leica −2065 supercut) and stained with 0.1% toluidine blue, pH 6.4. Static parameters of bone formation and resorption were measured in a defined area between 181 and 725 µm from the growth plate using an OsteoMeasure morphometry system (Osteometrics, Atlanta, GA) [55]. For dynamic histomorphometry, mineralizing surface per bone surface and mineral apposition rate was measured in unstained 10 µm sections under ultraviolet light, using a B-2A set long pass filter consisting of an excitation filter ranging from 450 to 490 nm, a barrier filter at 515 nm, and a dichromatic mirror at 500 nm. The terminology and units used are those recommended by the Histomorphometry Nomenclature Committee of the American Society for Bone and Mineral Research [56].

### Statistical analysis

The outcome measurements were expressed as means ± standard deviation (SD) or standard error of the mean (SEM). To compare the outcome mean measurements among the different groups, we used one-way ANOVA with Tukey’s post-hoc test or two-way ANOVA with Sidak multiple comparison test. For comparison between the two groups, the student t-test was used. All statistical tests were performed using the SPSS 23 software (IBM Corp) and Prism (GraphPad Software, v8). *p* values of less than 0.05 were considered statistically significant. *p < 0.05, **p < 0.005, ***p < 0.0005, ****p < 0.0001. ns, not significant.

## Supplementary Material

Final_Supplementary_Methods_and_Figures_revised_R6.docx

## Data Availability

The data set that substantiates the findings of this study is obtainable from the corresponding author upon receiving a reasonable request.
