# Supplementary material for: Bisphosphonate zoledronic acid blocks secretory autophagy and inhibits bone resorptive functions in osteoclasts
Source: Autophagy. 2026 Jun 28;22(11):3144–56. doi: 10.1080/15548627.2026.2691880 (PMC13712003; doi:10.1080/15548627.2026.2691880)
Supplement: Supplementary file 1 [file KAUP_A_2691880_SM5753.docx]

**Supplementary Materials and Methods**

**Bisphosphonate zoledronic acid blocks secretory autophagy and inhibits bone resorptive functions in osteoclasts**

Sol Kim^a,b,c^, Vadim Goldshteyn^a,b^, Atsushi Arai^a,d^, Eun-Bin Bae^a,b^, Julian Whitelegge^e^, Ki-Hyuk Shin^a,c^, No-Hee Park^a,f,g^, and Reuben H. Kim^a,b,c,f^

^a^The Shapiro Family Laboratory of Viral Oncology and Aging Research, UCLA School of Dentistry, Los Angeles, CA 90095, USA

^b^Division of Preventive and Restorative Sciences, UCLA School of Dentistry, Los Angeles, CA 90095, USA

^c^Section of Biosystems and Function, UCLA School of Dentistry, Los Angeles, CA 90095, USA

^d^Institute for Oral Science, Department of Orthodontics, Matsumoto Dental University, Nagano 399-0781, Japan

^e^The Pasarow Mass Spectrometry Laboratory, The Jane and Terry Semel Institute for Neuroscience and Human Behavior, UCLA David Geffen School of Medicine, Los Angeles, CA 90095, USA

^f^UCLA Jonsson Comprehensive Cancer Center, Los Angeles, CA 90095, USA

^g^UCLA David Geffen School of Medicine, Los Angeles, CA 90095, USA

Contact:

Sol Kim SolKim@dentistry.ucla.edu UCLA School of Dentistry, Center for the Health Sciences, Room 43-033, 10833 Le Conte Ave, Los Angeles, CA90095, USA

Reuben H. Kim rkim@dentistry.ucla.edu UCLA School of Dentistry, Center for the Health Sciences, Room 43-009, 10833 Le Conte Ave, Los Angeles, CA90095, USA

**Supplementary Materials and Methods**

***Bottom-up Proteomics via nano-LC-ESI-MS/MS***

Cells & lysis: BMMs cultured in CSF1/M-CSF alone were collected at day 0 as a basal control. For osteoclast differentiation, BMMs were differentiated with CSF1/M-CSF and TNFSF11/RANKL and treated with either vehicle (CTL) or zoledronate (ZOL) for the duration of culture. The CTL and ZOL groups were harvested on day 2 and day 4. Cell pellets were lysed in 180 µL buffer (12 mM sodium lauroyl sarcosinate; Millipore-Sigma, 1614374, 0.5% sodium deoxycholate; Millipore-Sigma, 30979, 50 mM TEAB ; Millipore-Sigma, 241059), bath-sonicated (10 min, Bioruptor Pico), and heated (95°C, 5 min). Protein was quantified by Micro-BCA (ThermoScientific, 23235); samples were adjusted to 0.5 mg/mL.

Reduction, alkylation, digestion: Aliquots (100 µL; 50 µg protein) were reduced with TCEP (10 µL, 55 mM in 50 mM TEAB; 30 min, 37 °C) and alkylated with chloroacetamide (10 µL, 120 mM; 30 min, RT, dark). Mixtures were diluted 5× with 50 mM TEAB and digested overnight with sequencing-grade trypsin (1 µg in 10 µL TEAB; ~1:50 w/w).

Phase transfer clean-up: An equal volume of ethyl acetate/TFA (100:1, v/v) was added, vortexed 5 min, and centrifuged (13,000 × g, 5 min). The upper organic layer was discarded; the aqueous phase was dried in a vacuum concentrator.

StageTip desalting (modified Rappsilber): Dried peptides were reconstituted in Solvent A (H₂O/ACN/TFA 98:2:0.1, 100 µL), loaded onto C18 StageTips (3M Empore) pre-conditioned with MeOH (20 µL), Solvent B (ACN/H₂O/TFA 80:20:0.1, 20 µL), and Solvent A (20 µL). After washing with Solvent A (20 µL), peptides were eluted with Solvent B (40 µL) and dried.

TMT labeling & pooling: Peptides were labeled with TMT-6plex (per manufacturer), quantified (Pierce peptide assay), and pooled to equalize loading (17 µg per condition; 102 µg total). The pooled sample was desalted again by StageTip and dried.

High-pH RP fractionation: Peptides were reconstituted in Solvent C (H₂O/ACN, 98:2 with 10 mM ammonium bicarbonate, pH 10; 5 µL). An aliquot (2.7 µL) was injected onto a ZORBAX 300 Extend-C18 column (0.3 × 150 mm, 3.5 µm) on an Agilent 1260 system at 6 µL/min. A linear gradient of Solvent D (ACN/H₂O 80:20 with 10 mM ammonium bicarbonate, pH 10) was run (0–60% over 65 min), fractions were collected into a 96-well plate preloaded with 20 µL of 5% formic acid, then concatenated to 12 fractions, desalted (StageTip), and dried.

NanoLC-MS/MS: Fractions were reconstituted in Solvent E (H₂O/ACN/FA 98:2:0.1; 10 µL) and 5 µL were injected onto a C18 nano column (AcuTech, 150 µm ID × 20 cm, 1.8 µm) on an Eksigent NanoLC-2D at 500 nL/min. A low-pH gradient to Solvent F (ACN/H₂O/FA 98:2:0.1) was applied (0–24% over 144 min; then 24–40% to 162 min; wash 80% to 174 min; re-equilibration).

Mass spectrometry: A Q Exactive Plus (Thermo) with nanospray source acquired data-dependent spectra: MS1 m/z 350–1700, resolution 70,000 (at m/z 200), AGC 3e6, max IT 50 ms; top-10 HCD MS/MS with 1.2 Th isolation, NCE 32, dynamic exclusion 30 s, resolution 35,000, AGC 1e5, max IT 100 ms; charge states ≥2 selected.

Data analysis: Raw files were processed in *Proteome Discoverer 2.2* for peptide/protein identification and TMT-based relative quantification.

Peptide micro-purification, enrichment, pre-fractionation, and storage using C18 StageTips followed Rappsilber et al. [1], with minor modifications.

**Proteomics data analysis.**

Reporter channels were mapped to time points as follows: Light = day 0 (CSF1/M-CSF only baseline), Medium = day 2, Heavy = day 4. Treatment groups were defined as CTL (untreated) and ZOL (treated). For each biological replicate and treatment, peptide/protein intensities were normalized to the day-0 baseline by computing day-2 change = Medium/Light (M/L) and day-4 change = Heavy/Light (H/L). Thus, for example, “CTL day 2” denotes the untreated M/L ratio, and “ZOL day 2” denotes the treated M/L ratio; analogous notation applies for day 4 (H/L). Results are reported as fold changes relative to day 0 within each treatment condition.

**Table S1.** List of oligonucleotide primers.

| Purpose | Primer Name | Sequence | Source | Size (bp) |
| --- | --- | --- | --- | --- |
| *Ctsk* Cre genotyping | *CtsK*-Cre | TTA TTC CTT CCG CCA GGA TG | [2] | Cre: 350 |
|  | *CtsK*-Cre | TAG TTT TTA CTG CCA GAC CG | T. Nakamura, 2007 |  |
| *Rab7* flox genotyping | *Rab7* F | GGC AAG GCA AGC ACT ACT GT | Jackson Lab, 16507 | WT: 625 |
|  | *Rab7* R | CAA ACT CAC TCA CTC CTA AAT GG | Jackson Lab, 16508 | flox: 700 |
| Mouse *Becn1* mRNA | *Becn1* F | AGGATGGTGTCTCTCGAAGATT |  | N/A |
|  | *Becn1* R | GATCAGAGTGAAGCTATTAGCACTTTC |  | N/A |
| Mouse *Sqstm1* mRNA | *Sqstm1* F | AGACCCCTCACAGGAAGGAC |  | N/A |
|  | *Sqstm1* R | CATCTGGGAGAGGGACTCAA |  | N/A |
| Mouse *Lc3* mRNA | *LC3* F | CCCCACCAAGATCCCAGT |  | N/A |
|  | *LC3* R | CGCTCATGTTCACGTGGT |  | N/A |
| Mouse *Atg4b* mRNA | *Atg4b* F | CCTTGGCTGTTCACATAGCA |  | N/A |
|  | *Atg4b* R | TGGCCCTGCATAACCTTCT |  | N/A |
| Mouse *Atg5* mRNA | *Atg5* F | AAGTCTGTCCTTCCGCAGTC |  | N/A |
|  | *Atg5* R | TGAAGAAAGTTATCTGGGTAGCTCA |  | N/A |
| Mouse *Cre* mRNA | *Cre* F | GCGGTCTGGCAGTAAAAACTATC |  | N/A |
|  | *Cre* R | GTGAAACAGCATTGCTGTCACTT |  | N/A |
| Mouse *Acp5* mRNA | *Acp5* F | CGTCTCTGCACAGATTGCAT |  | N/A |
|  | *Acp5* R | AAGCGCAAACGGTAGTAAGG |  | N/A |

**Figure S1.** ZOL does not trigger apoptosis at lower doses while diminishing autophagosome-lysosome fusion during osteoclast differentiation. Bone marrow-derived macrophages (BMMs) were treated with low doses of ZOL (1 and 5 µM) for 3 days. (**A**) Flow cytometry analysis for ANXA5 (annexin V). (**B**) Quantification of the flow cytometry analysis. (**C**) Western blotting against CASP3 (caspase 3). (**D**) Fluorescent staining for MAP1LC3/LC3 (red) and LAMP1 (green) visualized using confocal microscopy, with nuclear counterstaining (DAPI, blue). (**E**) Quantification of MAP1LC3/LC3-LAMP1 colocalization. More than 50 cells were counted in each condition. Graphs show the mean ± SD. ****p < 0.0001. Statistical analysis was performed using Unpaired t-test. (**F**) Fluorescent staining for MAP1LC3/LC3 (green) and RAB7 (red) was visualized using confocal microscopy. Nuclear counterstaining (DAPI, blue). Scale bars: 50 μm. (**G**) Colocalization profile.

**Figure S2.** F-actin formation of osteoclasts on PBS- or ZOL-treated dentin. (**A**) Dentin slices were pretreated with PBS or zoledronate (ZOL), then seeded with osteoclast precursors and differentiated with CSF1/M-CSF and TNFSF11/RANKL. Cells were fixed and stained with phalloidin (F-actin, green) and DAPI (nuclei, blue). Confocal images show intact actin rings on both PBS- and ZOL-treated dentin. (**B**) Z-stack orthogonal views (XZ/YZ) corresponding to the analysis in Fig. 3F, illustrating circumferential F-actin at the cell–dentin interface in both conditions. Scale bars: 10 µm.

**Figure S3.** Proteomic analysis in differentiating osteoclasts treated with or without ZOL. (**A**) Schematic of the experimental workflow: BMMs were stimulated with CSF1/M-CSF and TNFSF11/RANKL in the presence or absence of zoledronate (ZOL), followed by protein identification using mass spectrometry. (**B**) Volcano plot showing differentially expressed proteins between TNFSF11/RANKL and TNFSF11/RANKL + ZOL groups. A total of 377 proteins were significantly upregulated (red) and 282 downregulated (blue) in the TNFSF11/RANKL + ZOL condition. Gray dots represent non-significant changes. (**C**) GO enrichment analysis of upregulated proteins in the TNFSF11/RANKL + ZOL group. (**D**) GO enrichment analysis of downregulated in the TNFSF11/RANKL + ZOL group. (**E**) KEGG pathway enrichment of downregulated proteins, showing significant enrichment in the lysosome, phagosome, endocytosis, and RAS signaling pathways in the TNFSF11/RANKL + ZOL. (**F**) Heatmap showing expression patterns of selected RAB family and trafficking-related proteins.

**Figure S4.** ZOL does not alter RAB5 or RHOA post-modification during osteoclastogenesis. Primary BMMs were differentiated with CSF1/M-CSF and TNFSF11/RANKL for the indicated days (0–5) in the absence or presence of ZOL (1 µM). Whole-cell lysates were immunoblotted for RAB5 and RHOA with ACTB/β-ACTIN.

**Figure S5.** PCR genotyping of *rab7* conditional knockout mice and sex-stratified μCT and histomorphometric analysis in 8-week-old *rab7* cKO mice. (**A**) Tail DNA was analyzed by PCR with two assays. Top gel: *Rab7* floxed allele (fl) generates a 700-bp band; wild-type (+) generates a 625-bp band. Bottom gel: *Ctsk-Cre* transgene (350 bp) with endogenous *Ctsk* control (135 bp). Lanes are labeled for *Rab7* genotype (f/f, f/+, +/+) and for Cre status. Mice used as WT lack Cre (*Rab7^f/f^; Ctsk^+/+^*), whereas *rab7* cKO mice are *rab7^f/f^; Ctsk^Cre/+^.* Representative gels are shown.

(**B**–**E**) Somatic measures: body weight, femur length, spleen weight, and spleen/body-weight ratio.

(**F**–**I**) Distal femur μCT: BV/TV, trabecular number (Tb.N), trabecular separation (Tb.Sp), and cortical thickness (Ct.Th). Sample sizes for (**B**–**I**): *Rab7* WT: female, n=13; male, n=9. *rab7* cKO: female, n=11; male, n=8. (**J** and **K**) Osteoblast counts: total N.Ob and N.Ob per bone perimeter (N.Ob/B.Pm). (**L** & **M**) Osteoclast counts: total N.Oc and N.Oc per bone perimeter (N.Oc/B.Pm). Sample sizes for (**J**–**M**): *rab7* WT: female, n=8; male, n=7*. rab7* cKO: female, n=8; male, n=6.

(**N**) Paired values for individual animals illustrate the shift toward higher P1NP and lower CTX-I. (**O**) P1NP and CTX-I are inversely correlated (Pearson *r = -0.5852, p = 0.0173*). This supplementary figure supports the serum findings summarized in Fig. 5J and K. Graphs show the mean ± SEM. *p < 0.05, ***p < 0.0005, ****p < 0.0001. n.s., not significant. Two-way ANOVA with Sidak multiple comparison test (B-N).

**Figure S6.** *rab7* cKO osteopetrotic phenotype across sites and ages. (**A**) Representative Toluidine blue-stained sections of the L3 vertebra from 8-week-old *Rab7* WT and *rab7* cKO females. Scale bar: 100 μm. (**B**–**D**) Static parameters from the trabecular compartment: bone volume/tissue volume (BV/TV), trabecular number (Tb.N), and trabecular separation (Tb.Sp). Sample sizes for (**A**–**D**): *Rab7* WT n = 10, *rab7* cKO n = 8 (females). (**E**) Representative μCT of distal femur from 6-month-old mice. Top: longitudinal views; white arrows mark the plane of the trabecular cross-sections shown below. (**F**–**H**) Somatic measures: body weight, spleen weight, and spleen:body-weight ratio. (**I**) Femur length. (**J**) Trabecular bone volume/tissue volume (BV/TV). Sample sizes for (**F**–**J**): *Rab7* WT n = 5, *rab7* cKO n = 4 (males). (**K**) Toluidine blue-stained sections of the distal femoral metaphysis from 8-week-old *rab7* WT and *rab7* cKO mice. Scale bars: 100 μm. (**L**) Quantification of adipocyte number per marrow area (mm²). Sample sizes: WT: female n=10, male n=5; cKO: female n=9, male n=5. Points = individual mice. Graphs show the mean ± SEM. **p < 0.005, ***p < 0.0005, ****p < 0.0001. n.s., not significant. Unpaired t-test (B-D and F-J). Two-way ANOVA with Sidak multiple comparison test (L).

**Figure S7.** Dynamic histomorphometry of femur and L3 vertebra in *Rab7* WT and *rab7* cKO. (**A**) Representative double calcein labeling images for the trabecular bones from the femur of *Rab7* WT and *rab7* cKO mice. (**B**-**D**) Quantitative analyses of Mineral apposition rate (MAR), bone formation rate per bone surface (BFR/BS), and Mineralized surface per bone surface (MS/BS) (sex-pooled, *Rab7* WT: n=15, *rab7* cKO: n=14). (**E**) Representative calcein double labeling images for the trabecular bones from the vertebrae (L3) of *Rab7* WT and *rab7* cKO mice. (**F**–**H**) Dynamic indices: Quantitative analyses of Mineral apposition rate (MAR), bone formation rate per bone surface (BFR/BS), and Mineralized surface per bone surface (MS/BS). Each point is an individual mouse (female, *Rab7* WT n = 10, *rab7* cKO n = 8). Scale bars: 100 μm. Graphs show the mean ± SEM. *p < 0.05, ***p < 0.0005 ****p < 0.0001. ns, not significant. Unpaired t-test.

**Data S1.** Uncropped western blots corresponding to Figure 1A

LC3-I (16~18 kDa)

LC3-II (14~16 kDa)

15

10

GAPDH (37 kDa)

**Data S2.** Uncropped western blots corresponding to Figure 1B

LC3-I (16~18 kDa)

LC3-II (14~16 kDa)

GAPDH (37 kDa)

**Data S3.** Uncropped western blots corresponding to Figure 1C

LC3-I (16~18 kDa)

LC3-II (14~16 kDa)

ZOL

GAPDH

10

15

More exposed

LC3-I (16~18 kDa)

LC3-II (14~16 kDa)

ZOL

10

15

**Data S4.** Uncropped western blots corresponding to Figure 1E

SQSTM1/p62 (~62 kDa)

BECN1 (~52 kDa)

GAPDH (37 kDa)

ATG4B (~44 kDa)

**Data S5.** Uncropped western blots corresponding to Figure 1E

BECN1 (~52 kDa)

SQSTM1/p62 (~62 kDa)

GAPDH (37 kDa)

ATG4B (~44 kDa)

**Data S6.** Uncropped western blots corresponding to Figure 2A

**ACTB**

**(42 kDa)**

**Data S7.** Uncropped western blots corresponding to Figure 2B

**Cleaved CTSK**

**(~27 kDa)**

**Pro CTSK**

**(~40 kDa)**

**Data S8.** Uncropped western blots corresponding to Figure 2C

**GAPDH**

**(~37 kDa)**

**Pro MMP9 (~95-98 kDa)**

**Cleaved MMP9 (~86 kDa)**

**ACTB**

**(42 kDa)**

**Data S9.** Uncropped western blots corresponding to Figure 2D

**Cleaved ACP5/TRAP**

**(20~23 and 16 kDa)**

**Pro-ACP5/TRAP**

**(~37 kDa)**

**GAPDH**

**(~37 kDa)**

**Data S10.** Uncropped western blots corresponding to Figure 2H

**ACTB**

**(42 kDa)**

**Data S11.** Uncropped western blots corresponding to Figure 4D.

Fig. 4e

**Data S12.** Uncropped western blots corresponding to Figure 4H

RAB7

(~23 kDa)

GAPDH

(~37 kDa)

**Data S13.** Uncropped western blots corresponding to Figure 4I

RAB7 (~23 kDa)

GAPDH

(~37 kDa)

**Data S14.** Uncropped western blots corresponding to Figure 4J

SQSTM1/p62 (~62 kDa)

RAB7 (~23 kDa)

**Cleaved CTSK (~27kDa)**

**Pro-CTSK (~40 kDa)**

GAPDH

(~37 kDa)

**Data S15.** Uncropped western blots corresponding to Figure 6B

**Data S16.** Uncropped western blots corresponding to Figure 6H

**Data S17.** Uncropped western blots corresponding to Figure 6I.

**Data S18.** Uncropped western blots corresponding to Figure 6J

**Data S19.** Uncropped western blots corresponding to Figure S1C

Cleaved CASP3

(~17 kDa)

CASP3 (~32 kDa)

GAPDH

**Data S20.** Uncropped western blots corresponding to Figure S6.

(The same membrane as Fig. 2A)

**ACTB**

**(42 kDa)**

**Reference**

[1]	Rappsilber J, Mann M, Ishihama Y. Protocol for micro-purification, enrichment, pre-fractionation and storage of peptides for proteomics using StageTips. Nat Protoc. 2007;2(8):1896-906.

[2]	Nakamura T, Imai Y, Matsumoto T, et al. Estrogen prevents bone loss via estrogen receptor alpha and induction of Fas ligand in osteoclasts. Cell. 2007;130(5):811-23.
